# *olf413* an octopamine biogenesis pathway gene is required for axon growth and pathfinding during embryonic nervous system development in *Drosophila melanogaster*

**DOI:** 10.1186/s13104-024-06700-3

**Published:** 2024-02-07

**Authors:** Ravindrakumar Ramya, Chikkate Ramakrishnappa Venkatesh, Baragur Venkatanarayanasetty Shyamala

**Affiliations:** Developmental Genetics Laboratory, Department of Studies in Zoology, University of Mysore, Mysuru, 570006 India

**Keywords:** *Drosophila*, *olf413*, Octopamine, Dopamine, *TβH*, Axonal pathfinding, Axonal growth, Nervous system development

## Abstract

**Objective:**

Neurotransmitters have been extensively studied as neural communication molecules. Genetic associations discovered, and indirect intervention studies in Humans and mammals have led to a general proposition that neurotransmitters have a role in structuring of neuronal network during development. *olf413* is a *Drosophila* gene annotated as coding for dopamine beta-monooxygenase enzyme with a predicted function in octopaminergic pathway. The biological function of this gene is very little worked out. In this study we investigate the requirement of *olf413* gene function for octopamine biogenesis and developmental patterning of embryonic nervous system.

**Result:**

In our study we have used the newly characterized neuronal specific allele *olf413*^*SG1.1*^, and the gene disruption strain *olf413*^*MI02014*^ to dissect out the function of *olf413*. *olf413* has an enhancer activity as depicted by reporter GFP expression, in the embryonic ventral nerve cord, peripheral nervous system and the somatic muscle bundles. Homozygous loss of function mutants show reduced levels of octopamine, and this finding supports the proposed function of the gene in octopamine biogenesis. Further, loss of function of *olf413* causes embryonic lethality. FasII staining of these embryos reveal a range of phenotypes in the central and peripheral motor nerves, featuring axonal growth, pathfinding, branching and misrouting defects. Our findings are important as they implicate a key functional requirement of this gene in precise axonal patterning events, a novel developmental role imparted for an octopamine biosynthesis pathway gene in structuring of embryonic nervous system.

## Introduction

Neurotransmitters are classical communication molecules extensively studied for their synaptic function mediating transmission. Human and mammalian studies which associate many of the major neurotransmitters to neurodevelopmental disorders implicate neurotransmitters to have potential roles beyond synaptic communication, in neurodevelopment [1]. The enormous size and complexity of mammalian brain makes it difficult to visualize the developmental deformities at a finer resolution in terms of cellular processes and axonal tracts of neurons. *Drosophila,* which is capable of performing complex activities has a relatively several folds lesser number of neurons in their brain with simpler and definable circuits [2]. Further flies as model system offer the most tractable genetic toolkit which enables the analysis of genes for their functions in a context dependent manner [3–8]. Octopamine is a neurotransmitter similar to vertebrate nor adrenaline, controls aggression, sleep, appetite, feeding, courtship etc. in *Drosophila*, as understood through studies on mutants defective for enzyme Tyramine β-Hydroxylase [9]. Here we report an intriguing finding that a paralogous gene *olf413*, predicted to be involved in octopamine biogenesis is essential for developmental patterning of embryonic nervous system.

SG1.1 is a P-*GAL4* enhancer trap strain isolated in our earlier screen for genes with expression in the nervous system [10]. This strain carries single P-*GAL4* insertion on the third chromosome which is homozygous lethal. The strain shows enhancer activity in clusters of neurons in the suboesophageal ganglion (SOG), superior protocerebrum, central brain (CB) region and ventral ganglion (VG) of the adult brain [11, 12]. The expression of the reporter gene in the pupal brain in a temporally regulated cyclical pattern prompted us to characterize the native gene at the site of P-*GAL4* insertion in SG1.1 strain. Here we report the molecular mapping of the P-*GAL4* insertion to 1.9 bp upstream of the transcription start site of the identified native gene *olf413,* as annotated in Berkeley Drosophila Genome Project (BDGP) [13]. The reporter gene expression comparisons and complementation test with a null allele of *olf413* confirmed SG1.1 P-*GAL4* insertion as an allele of *olf413*. Gene *olf413*, annotated as CG12673 has been predicted to code for a protein paralogous to *Tβh* having a role as an enzyme in octopamine biogenesis [14]. Biological function of *olf413* has been very little worked out, except in a few genome-wide association (GWA) screens, food preference and motor behavior analysis [15–19]. This study shows that *olf413* mutants have decreased octopamine levels and the loss of function mutant embryos show severe disruptions in motor nerves of ventral nerve cord (VNC) and their peripheral motor axon projections. Our observations discretely demonstrate in vivo*,* the critical requirement of *olf413* function in establishing precisely patterned neuronal network during embryonic development.

## Methods

### *Drosophila* stocks

The following fly stocks were used: Oregon-K (Drosophila Stock Centre, University of Mysore), UAS-*GFP* on III chromosome (National Centre for Biological Sciences, Bengaluru), SG1.1/*TM3Sb* (our lab), Gene disruption strain *olf413*^*MI02014*^*/TM3Sb* (#77717, Bloomington Drosophila Stock Centre (BDSC)) [20].

### Molecular localization of P-*GAL4* insertion

Inverse PCR was carried out using T7 and T3 primers on self-ligated Sac1 and Pst1 digests of SG1.1 genomic DNA. The genomic fragment flanking the P- insertion was sequenced. The flanking genomic sequence mapping was done using Basic Local Alignment Search Tool (BLAST) search against *Drosophila melanogaster* genomic sequence to obtain the precise position of P-*GAL4* insertion.

### Lethality test

To decipher the exact stage of lethality in homozygous SG1.1 strain, embryos were collected on 2% sucrose agar medium at 22 ℃ overnight. 1000 embryos were transferred to 20 vials containing normal wheat cream agar medium with 50 embryos in each vial. The number of live and dead individuals were scored and recorded at each stage (larva, pupa and adult).

To check embryonic lethality, embryos collected were incubated at 25 ℃ incubator for nearly 48–50 h. The number of unhatched and hatched embryos were tabulated. The unhatched embryos of genotype SG1.1-*GAL4* and *olf413*^*MI02014*^, which had completed embryogenesis and were with visible mouth hook were imaged in halocarbon oil under Bright field microscope (ZeissAXIO Imager A2).

### Complementation test

Virgin females from SG1.1-*GAL4*/*TM3Sb* were crossed to *olf413*^*MI02014*^*/TM3Sb* males, and the F1 progeny embryos were collected and grown as explained in lethality test. The number of F1 adult flies eclosed from 50 × 20 replicates were counted and recorded.

### Analysis of reporter gene activity

UAS-*GFP*; SG1.1/*TM3Sb* stock was generated and used for analysis of GFP expression. *olf413*^*MI02014*^*-GAL4* virgin females were crossed with UAS-*GFP* (on III chromosome) males, to obtain *olf413*^*MI02014*^*-GAL4*/UAS-*GFP* individuals. The embryos collected were dechorionated in 50% sodium hypochlorite solution, rinsed with water, mounted in halocarbon oil and the GFP expression was documented using confocal laser scanning microscope (Zeiss LSM 710). The Z sections were taken at every 1 µm interval. The 3D projections of Z-Stacks were obtained.

Staged larval and pupal brains were dissected in phosphate buffered saline (PBS) and fixed in 4% paraformaldehyde (PF) fixative in PBS, for 20–30 min at room temperature. The fixed brains were washed in PBS and 0.05% PBTx and mounted in vectashield for imaging.

### Immunohistochemistry

15–18 h old embryos selected from an overnight collection of Oregon-K, SG1.1/*TM3Sb* and *olf413*^*MI02014*^ stocks were anaysed. The embryos were dechorionated and fixed in 3.7% formaldehyde in PBS with equal volume of n-heptane (1:1) solution for 20 min, devitenalized by 2–3 methanol washes, followed by washes with absolute alcohol. The embryos were rehydrated with grades of alcohol and 0.1% PBTx (1:2, 1:1, 0.1%PBTx) prior to Antibody staining and processed further. Third instar larval brains were dissected in PBS, fixed in 4% PF for 25 min at room temperature, washed with 0.05% PBTx and processed for antibody staining. Antibody staining for both embryos and larval brains was carried out as described in Rohith and Shyamala [21], with minor changes. Primary antibodies used were mouse anti-Fasciclin II (1:10, 1D4, DSHB) for embryos, mouse anti-Repo (DSHB; 1:10) and mouse anti-Elav (DSHB; 1:10) for larval brains. Secondary antibodies used were Goat Anti-Mouse IgG CF™ 488A (1:200; Sigma Aldrich), Goat Alexa fluor 647 anti-mouse IgG (Invitrogen; 1:500). The embryos and brains were mounted in vectashield and imaged with confocal laser scanning microscope (Zeiss LSM 710).

### Octopamine quantification

LC–MS/MS analysis was carried out for control (Oregon-K) and mutant (*olf413*^*MI02014*^ homozygous survivors). 10 heads of just eclosed males and females in equal ratio, were homogenised with 50 μl PBS and 50 μl of 0.1% Formic acid in Acetonitrile (ACN), and centrifuged at 10,000 rpm for 10 min at 5 °C. The supernatant was collected, and stored at − 20 °C until analysis. Octopamine levels were measured with HPLC—Shimadzhu LC Prominence 20AT, and Mass Spectrophotometer—AB Sciex, 4000 equipped with a C18, 50*4.6 mm, 4-micron column. The graph represents a mean of three independent experimental replicates for each group.

### Statistics

The statistical analysis was performed using SPSS software (Version 22). The octopamine quantity is presented as mean ± SEM. One-way ANOVA and Tukey's post-hoc honestly significant difference test were applied to compare between the control and mutant groups.

## Results

### SG1.1 P-*GAL4* insertion is an allele of *olf413*

**Fig. 1:**
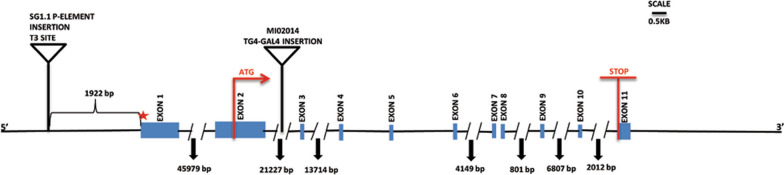
Mapping of insertion site of P-*GAL4* element in *olf413*^*SG1.1*^ allele of *olf413* gene. The figure depicts the P-*GAL4* insertion site in *olf413*^*SG1.1*^ and the gene disruption strain, *olf413*^*MI02014*^ on a 5’ to 3’ directed *olf413* gene span. The rectangular boxes represent the exons. The thin lines indicate the introns. The numbers along each intron represents the length of the respective intron. SG1.1 P-*GAL4* element is inserted at 1922 bp upstream of the transcriptional start site of *olf413* (red star). The insertion site of Trojan-*GAL4,* and the position of start codon (ATG) and Stop codon in *olf413* are marked on the basis of BDGP genome data [13, 20]

**Fig. 2:**
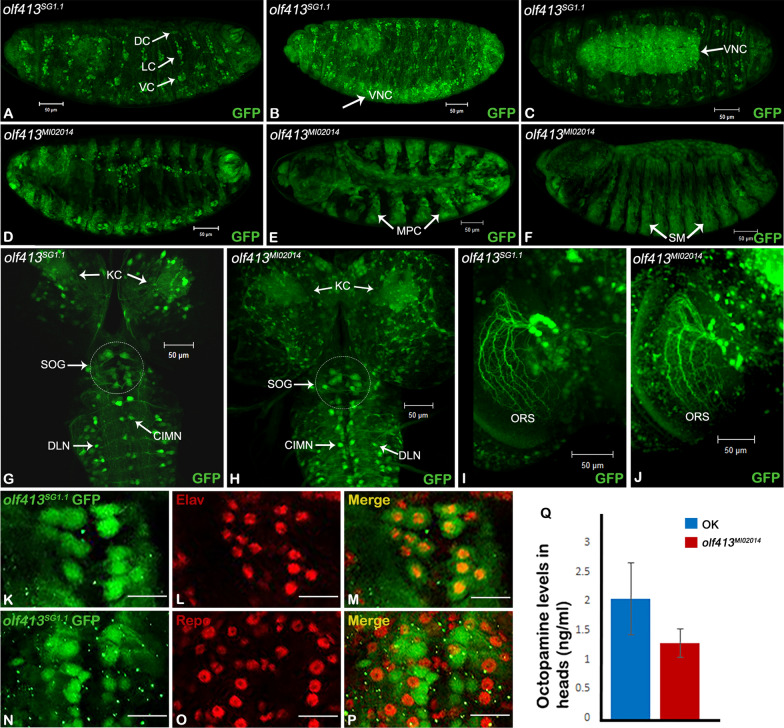
Enhancer activity pattern in *olf413*^*SG1.1*^ enhancer trap strain and *olf413*^*MI02014*^ as depicted by reporter GFP expression. UAS-*GFP* strain was crossed with the two *olf413* strains and the reporter GFP expression was analysed. **A** Embryo (stage 16) of *olf413*^*SG1.1*^ shows an expression in the ventral nerve cord region, in ventral (VC), lateral (LC) and dorsal (DC) neuronal clusters of nervous system. **B** and **C** Stage 12 and stage 17 embryos of *olf413*^*SG1.1*^ respectively showing the strong expression of GFP in the developing ventral nerve cord (VNC). **D** Stage 16 embryo of *olf413*^*MI02014*^*-GAL4/*UAS-*GFP* showing expression pattern similar to *olf413*^*SG1.1*^. E and **F** Stage 12 and stage 17 embryos of *olf413*^*MI02014*^ respectively with a very prominent reporter expression in the somatic muscle precursors (MPC) and Somatic muscle bundles (SM). **G** and **H** 72 h after larval hatched (ALH) (late 3rd instar larva) staged brain of *olf413*^*SG1.1*^ and *olf413*^*MI02014*^ strains respectively shows the reporter gene expression in clusters of neurons in central brain, suboesophageal ganglion (SOG) and Kenyon cells (KC). Ventral ganglion has the expression in centrointermedial neurons (CIMN) and the dorsolateral neurons (DLN) of individual segmental neuromeres. **I** and **J** Frontal view of adult brain (101 h APF) of *olf413*^*SG1.1*^ and *olf413*^*MI02014*^ respectively showing lobular plate region. The vertical system neurons of the optomotor responsive system [25] (ORS) shows strong enhanced activity in both the strains. All embryos have anterior to the left and the posterior to the right. **K**–**M** Brains at 72 h ALH, stained with Anti-Elav antibody (**L**, Red) marked with *olf413*^*SG1.1*^ GFP (**K**, green). **M** merged image showing that *olf413*^*SG1.1*^ cells are Elav positive. **N**–**P** Brains at 72 h ALH stained with Anti- Repo antibody (**O**, Red) and marked with *olf413*^*SG1.1*^ GFP (**N**, green). **P** merged image showing that *olf413*^*SG1.1*^ cells do not overlap with Repo positive cells. **Q** Represents the results of LC–MS/MS analysis for octopamine quantification. The bar graphs represent the mean ± SEM of octopamine levels (n = 3) quantified in head samples for homozygous *olf413*^*MI02014*^ with Oregon-K as control. **A**, **B**, **D**, **E**, **F** are lateral views, **C** is ventral view. Scale bar in Fig A-J represents 50 μm and in (**K**–**P**) represents 10 µm

Precise mapping of P-*GAL4* insertion in SG1.1 strain was done by Inverse PCR (Methods for details). Two independent restriction digests of SG1.1 genomic DNA using SacI and PstI, were processed for inverse PCR with T7 and T3 primers (Methods for details). The BLAST search of flanking genomic DNA maps P-*GAL4* insertion in this strain to 22,134,168th bp position on the left arm of the 3rd chromosome (79C). This insertion site is 1.9 kb upstream of the transcriptional start site of the annotated gene [13] CG12673 identified as *olf413* (Fig. 1). There are no other annotated protein coding transcripts up to a distance of about 53,370 bases from the site of P-insertion. There is an annotated long non coding RNA—lncRNA-CR45236 at a distance of 12.23 kb upstream of the insertion site whose molecular and biological function are not known. The reporter gene expression pattern as depicted by the expression of UAS-*GFP*, driven by SG1.1-*GAL4* was documented. The embryonic expression pattern of GFP (Fig. 2A) matches with that of the mRNA in situ hybridization pattern for CG12673 [22].

### SG1.1 strain has an enhancer activity which covers a subset of expression domains of *olf413* in embryonic and adult nervous system

Lee et al. have generated a gene disruption library of strains which have gene specific T2A-*GAL4* insertion in one of the introns of the target gene, thus will be expressed under the endogenous promoter of the gene in question. The transgenic construct also has a polyadenylation signal at 3’end of the *GAL4* coding sequence which will arrest transcription following the *GAL4* sequence. Thus it will code for a truncated protein for the target gene, and acts as a null/severe loss of function allele for the gene [20]. *olf413*^*MI02014*^ is a gene disruption strain from this library which has the T2A-*GAL4* insertion in the second intron after the translational start site for CG12673, the annotated gene for *olf413*. *olf413*^*MI02014*^ and SG1.1-*GAL4* strains were crossed to UAS-*GFP* and the *GAL4* driven reporter GFP expression was analyzed and compared. The reporter gene expression driven by SG1.1-*GAL4* is seen in the ventral (VC), lateral (LC) and dorsal clusters (DC) of neurons of the peripheral nervous system (PNS) in stage 16 embryo [23] (Fig. 2A). A similar pattern of enhancer activity is seen in *olf413*^*MI02014*^/UAS-*GFP* embryos (Fig. 2D). The two strains share an overall similarity in the pattern of reporter GFP expression in the larval brain (Fig. 2G and H). Clusters of neurons in the central brain, suboesophageal (SOG) and the ventral ganglion are strongly marked while Mushroom body neurons, the kenyon cells (KCs) show faint expression. Segmentally reiterated pairs of neurons along the neuromeres of the VG, which localize to the centrointermedial (CIMN) and the dorsolateral (DLN) compartments of the fasciclin II (FasII) landmark system [24], express reporter GFP. It is seen that the SG1.1 enhancer activity is restricted to fewer number of neurons as compared to that seen in case of *olf413*^*MI02014*^ in the respective domains of expression. Figure 2I and J show the lobula plate region of the adult brains of SG1.1/UAS-*GFP* and *olf413*^*MI02014*^/UAS-*GFP* individuals respectively. The dendritic arborization of the vertical system neurons (VS1, VS2, VS3) of the optomotor responsive system (ORS) [25] are strongly marked by the reporter GFP expression. Besides this shared enhancer activity, there are expression patterns which are distinct to the two strains. We see that the embryonic ventral nerve cord in SG1.1-*GAL4* is marked by reporter expression from stage 12, which becomes pronounced by stage 17 (Fig. 2B, C). The endogenous enhancer activity as in *olf413*^*MI02014*^, is seen in the developing somatic muscles. Reporter expression is seen in myoblast clusters of stage 12 embryo, and the dorsal and lateral somatic muscle (SM) bundles of late 17 stage embryo (Fig. 2E, F). This mesodermal specific enhancer activity is not seen in SG1.1-*GAL4* strain. The adult and larval expression patterns of SG1.1-*GAL4* enhancer trap strain recorded during our earlier screening had shown reporter expression restricted to the brain and ventral ganglion of the adult and the larva. Adult and the larval muscles, including other tissues do not show reporter expression [10, 11]. Further to confirm the neural or glial identity of these cells, we have here stained the SG1.1 *GAL4*-*GFP* larval brains with Anti-Elav (neuronal) and Anti-Repo (glial) marker antibodies. Figure 2K–P depicts the results. We can clearly see that all SG1.1-GFP expressing cells stain positive with anti-Elav antibody (Fig. 2K–M), whereas none of the GFP expressing cells co-localize with anti-Repo antibody (Fig. 2N–P). Thus, demonstrating clearly that, the P-*GAL4* insertion in SG1.1 marks a neuronal specific enhancer of *olf413*.

### SG1.1-*GAL4* insertion fails to complement lethality in-trans with *olf413*^*MI02014*^

Prompted by the fact that, P-*GAL4* insertion localizes at 1.9 kb upstream of *olf413* transcription start site, and the local enhancer activity pattern maps to a subset of endogenous enhancer activity domains of *olf413*, we subjected the two strains for complementation in-trans test. As mentioned earlier, SG1.1-*GAL4* insertion strain is homozygous lethal at embryonic stage. We checked the gene disruption strain *olf413*^*MI02014*^ and found that it also shows lethality at embryonic stage with very few escapers (3–4%). The results of the complementation test (Methods) are presented in Table 1. The transheterozygotes SG1.1-*GAL4*/*olf413*^*MI02014*^ showed a partial lethality of about 36.4%. This failure in complementation further confirmed that SG1.1-*GAL4* strain is a new allele of *olf413*. And here onwards we denote the strain as *olf413*^*SG1.1*^- a new allele of *olf413*.

**Table 1:** Complementation test- homozygous and in-trans lethality *olf413*^*SG1.1*^ and *olf413*^*MI02014*^ alleles

	Control Oregon-K	*olf413*^*SG1.1*^ (Homozygous)	*olf413*^*MI02014*^ (Homozygous)	*olf413*^*SG1.1*^*/olf413 *^*MI02014*^
Number of progeny expected	500	250	100	250
Number of progeny observed	434	0	4	159
% Lethality observed	13.2%	100%*	96%*	36.4%*

The table shows the lethality in percent of homozygotes *olf413*^*SG1.1*^-*GAL4*, homozygotes *olf413*^*MI02014*^ and transheterozygotes *olf413*^*SG1.1*^*/olf413*^*MI02014*^ counted as number of non-stubble flies eclosed (complementation test, methods for details) with Oregon-K flies as control

^*^This lethality was calculated by counting the number of non- stubble flies eclosed

### *olf413* loss of function mutants show reduced levels of octopamine

Based on the annotated protein product and the identified functional domains, the function of *olf413* gene product is predicted as dopamine beta-monooxygenase, an enzyme in octopamine/ norepinephrine biogenesis. In this context, we wanted to check if octopamine levels are affected in the loss of function mutants of *olf413*. The head samples from *olf413*^*MI02014*^ homozygous survivor adults were subjected to LC–MS/MS analysis for quantification of octopamine. Three sample runs for each trial set were carried out for the control and the mutant (Methods for details). The results are presented as bar graphs in Fig. 2Q. We find that, in all the trials, the mutant heads consistently showed reduced quantity of octopamine compared to that of the head samples from the control. The results strongly substantiate the predicted function of *olf413* in octopamine biosynthesis.

### *olf413* loss of function results in severe axonal growth, pathfinding and connectivity defects in embryonic nervous system

**Fig. 3:**
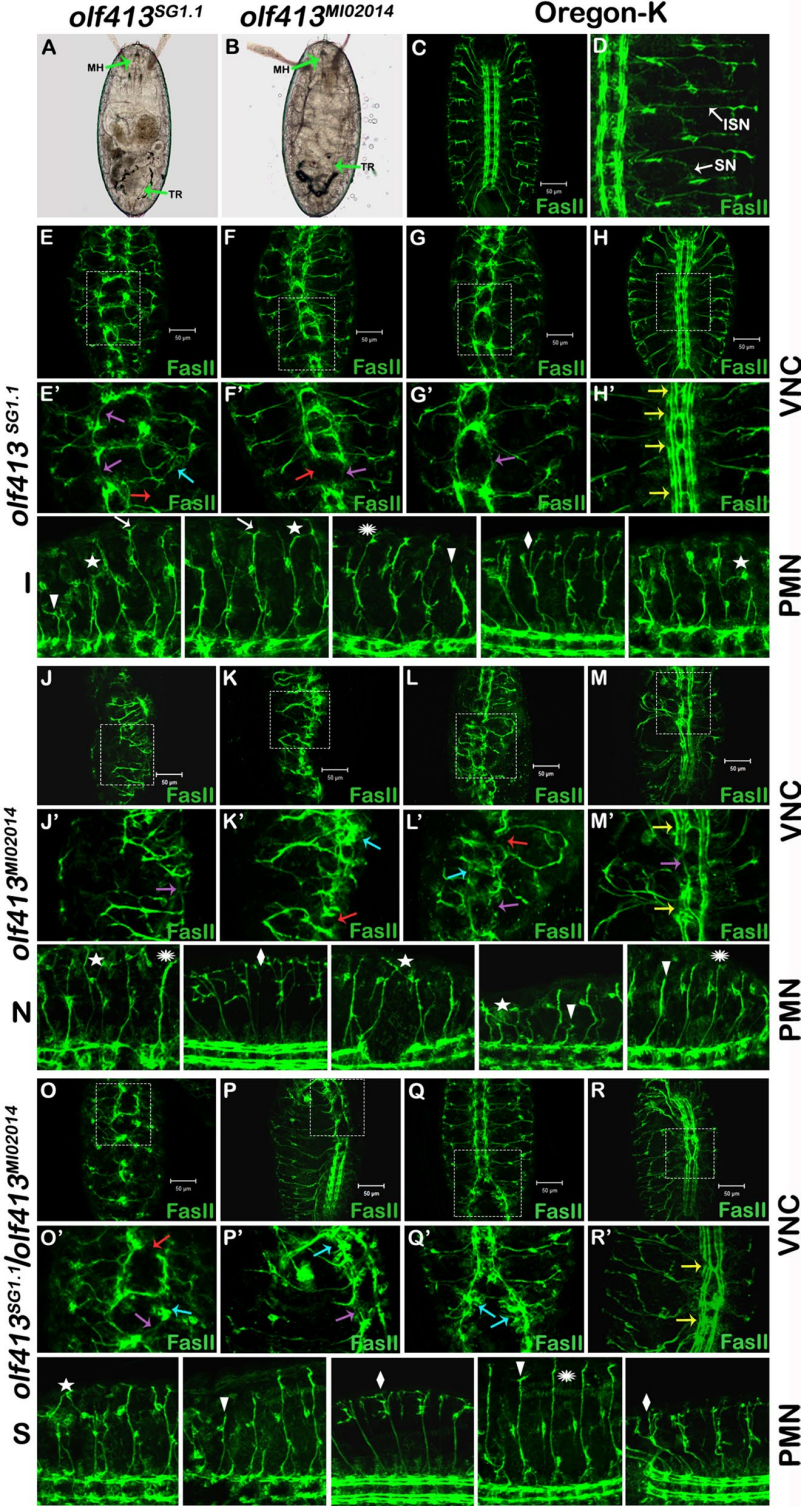
Central and peripheral motor neuron axon guidance defects in mutant *olf413*^*SG1.1*^, *olf413*^*MI02014*^ and trans-heterozygotes *olf413*^*SG1.1*^*/olf413*^*MI02014*^ embryos. **A** and **B** The bright field images of lethal embryos of *olf413*^*SG1.1*^ and *olf413*^*MI02014*^ homozygotes respectively showing the mouth hook (MH) and trachea (TR) completely formed (green arrows). **C–S** Embryos at stage 16–17 stained with Anti-Fasciclin II antibody (mAB-1D4) to label the axonal tracts of both ventral nerve cord and peripheral nerves. **C** and **D** are the Oregon-K embryos of stage 16–17, showing the wild type VNC and the peripheral motor nerves (PMN) respectively. The inter-segmental nerve (ISN) and segmental nerve (SN) are marked with white arrows. **E**–**I** Represent the VNC and the PMN of *olf413*^*SG1.1*^ homozygotes. **E** Most severe VNC and PNS deformities. **F** and **G** Moderate phenotype with axonal growth defect in VNC, disrupted and misrouted PMN. **H** Midline crossing of longitudinal tracts. **E’**–**H’** Enlarged view of marked region in (**E**–**H**). **I** Lateral view of peripheral projection nerves showing different growth and pathfinding defects. **J**–**N** Represent the VNC and PMN of *olf413*^*MI02014*^ homozygotes. **J**–**M** Most severe VNC and PNS deformities. **J’**–**M’** Enlarged view of marked region in (**J**–**M**). **N** Lateral view of peripheral projection nerves showing severe growth and pathfinding defects. **O**–**S** Represent the VNC and PMN of *olf413*^*SG1.1*^/*olf413*^*MI02014*^ transheterozygotes. **O** Most severe VNC and PNS deformities. **P** and **Q** moderate growth and pathfinding defects. **R** Mid line crossing of longitudinal tracts. **O’**–**R’** Enlarged view of marked region in (**O**–**R**). **S** Lateral view of peripheral projection nerves showing different growth and pathfinding defects. In all figures, arrows indicate breaks in VNC (red), axonal thinning and growth defect (purple), disordered or misrouted axons evading segmental boundaries (blue), and midline crossing of the longitudinal tracts (yellow). The peripheral motor projection defects are indicated by arrowheads- premature termination of growth, asterisks- fusion or fasciculation of ISN and SN into one nerve, rhomboid- excessive or abnormal branching of axons, star- misrouting of axons extending across the segmental boundaries, where the ISN of one segment fasciculate with the nearby ISN segment, and white arrow- de-fasciculation or branching at the ends of ISN. In the images showing VNC, the embryos are positioned with anterior to the top, whereas for the embryos showing PMN, they are positioned with anterior to the left. Scale bar represents 50 μm

The homozygous embryos of *olf413*^*SG1.1*^ as well as, that of *olf413*^*MI02014*^ when observed, revealed that they die at very late stage of embryonic development, with the mouth hook and trachea formed (Fig. 3A and B). Intriguingly, some of these embryos showed wriggling movements within the chorion and made attempts to hatch out, but failed to move out of the chorion. This observation was strongly indicative of a probable problem with the neuromuscular coordination and disability. Fasciclin II antibodies provide an effective tool to identify motor axon defects. We stained the late staged (16–17 stage) homozygous embryos with Anti-FasII antibody. The results are presented in Fig. 3C–S. Figure 3C and D represent wild type ventral nerve cord and peripheral motor nerves pattern. The *olf413*^*SG1.1*^ homozygous embryos showed grades of deformities in the longitudinal tracts of the ventral nerve cord and the peripheral motor nerves (Fig. 3E–I). Most severe phenotypes with an occurrence of 40% had highly disorganized ventral nerve cord and the peripheral motor projections (Fig. 3E). Embryos with moderate phenotypes had breaks in the VNC, axonal growth defects, disordered and misrouted axons evading segmental boundaries (Fig. 3F and G). The milder phenotypes presented frequent midline crossing of the longitudinal tracts (Fig. 3H). The enlarged views highlight these defects in detail (Fig. 3E’–H’). The inter segmental (ISN) and segmental (SN) motor projection neurons showed defects like fusion of ISN and SN nerves into single tract, misrouting of axons to cross the segmental boundaries. Excessive and abnormal branching, premature termination of growth before they reach their respective target muscles were commonly seen among motor axon projections (Fig. 3I).

The VNC and peripheral motor projection axon phenotypes seen in homozygous embryos of *olf413*^*MI02014*^ strain were relatively much more severe (Fig. 3J–N). This was expected as loss of function of *olf413* in this case is both in the neurons as well as in the somatic muscles. Highly disordered and messed up VNC with stunted and misrouted projection nerves featured the phenotypes with an occurrence of 56.2% (Fig. 3J–M). Detailed view of these defects are seen in Fig. 3J’–M’. Multiple defects found in peripheral motor projection nerves were comparable to that seen in *olf413*^*SG1.1*^ homozygotes (Fig. 3N). In summary, the phenotype observed demonstrates that the function of *olf413* is critically required for proper axonal growth, guidance, branching and target connectivity in both the VNC and peripheral motor projections during embryonic development. The transheterozygote *olf413*^*SG1.1*^*/olf413*^*MI02014*^ embryos stained with Anti-FasII antibodies showed similar range of longitudinal tract and motor axon pathfinding defects (Fig. 3O–R). The frequency of occurrence of the most severe phenotypes was 46.15%. Figure 3O’–R’ show enlarged views of the VNC defects. Figure 3S depicts various peripheral motor projection defects observed. All the axonal tract defects seen in the *olf413*^*SG1.1*^ and *olf413*^*MI02014*^ homozygotes were also seen in the transheterozygotes, though represented in lesser number of embryos. These results imply that the two strains fail to complement each other with respect to the axonal tract defects as well.

## Discussion

SG1.1, a P-*GAL4* strain was studied in detail for its molecular localization, enhancer activity pattern and genetic complementation with a putative candidate native gene *olf413*. Our experiments demonstrated that SG1.1-*GAL4* strain is a neuronal specific allele of *olf413* (CG12673). *olf413* has been annotated as a protein coding gene with predicted copper type II ascorbate-dependent monooxygenase domain, tyramine/dopamine beta- hydroxylase signature domains [26]. The biological functions of *olf413* have been little studied. It has been identified as an associated gene in a few GWA analysis studies carried out for psychostimulant drug preferences [15] and dietary dependent reduction in life span and starvation resistance [16, 17]. Recently studies have shown food preference, feeding and motor activity defects in *olf413* mutant adults [18, 19].

In the present study, we have used the gene disruption strain *olf413*^*MI02014*^ in conjunction with *olf413*^*SG1.1*^ to describe for the first time, the detailed expression pattern and role of the gene *olf413* in embryonic development and octopamine biogenesis. Our finding that the homozygous mutants show a decreased level of octopamine, strongly agrees with the predicted function of the gene in octopamine biogenesis. Analysis of lethal homozygous (*olf413*^*SG1.1*^, *olf413*^*MI02014*^) and transheterozygote (*olf413*^*SG1.1*^*/olf413*^*MI02014*^) by Anti-FasII antibody staining has revealed extremely severe to mild deformities in the embryonic ventral nerve cord and peripheral motor projection nerves. Relatively more distorted VNC and peripheral axonal phenotypes in *olf413*^*MI02014*^ embryos indicate that, the neuronal, as well as the muscle specific expression of the gene are required, and function in synergy with each other to facilitate axon growth and guidance to establish precise patterning of the neuronal tracts during embryonic development.

By DRSC integrative ortholog prediction tool (DIOPT) reports [27], *olf413* has been identified as a paralogue of *TβH* gene which codes for Tyramine β Hydroxylase, a key enzyme in octopamine biosynthesis pathway [28, 29]. Octopamine being a neurotransmitter and neuromodulator [30, 31] *TβH* null mutants which suffer octopamine deficits have been assayed for various behavioural phenotypes. TβH function has been implied in regulating aggression [32], courtship [33], sleep behavior [34], learning and memory [35], dietary response [36, 37] larval locomotion [38] and Tau pathogenicity in flies [39]. Nevertheless, so far in vivo embryonic expression and embryonic mutant phenotypes imparting a developmental role, have not been demonstrated for *TβH*. *TβH* null flies survive till adulthood with normal morphology but exhibit ovulation defect [14, 40]. While orthologous genes typically perform equivalent functions, paralogues in general evolve through subfunctionalization and subsequent neofunctionalization [41]. Here we have shown that *olf413*, a paralogue of *TβH*, has been deployed to perform a distinct role in the development of embryonic nervous system. Developmental role for neurotransmitters has long been implicated in Humans and other mammalian systems [1]. Studies have been carried out through pharmacological interventions using receptor blockers and antagonists in mammalian models for major neurotransmitters like serotonin [42–45], norepinephrine [46], dopamine [47], GABA [48, 49] and acetylcholine [50]. Genetic studies in Humans include classical chromosomal aberrations associations, and genome-wide association studies (GWAS) relating neurotransmitters to neurodevelopmental diseases [51, 52]. The probable structural connectivity disturbances in these studies have been suggested based on the behavioral and cognitive impairments and altered electrical recordings observed in the subjects. A few in vitro cell culture studies have shown that neurotransmitters administered in culture modulate axon growth and branching. [53, 54]. These studies show indirectly the non-synaptic, developmental roles of neurotransmitters in mammalian brain. The relative simplicity of the cellular content and the neuronal circuitry in *Drosophila* nervous system has allowed us to demonstrate in this study for the first time, the functional requirement of an octopamine biosynthesis pathway gene for precise axonal growth, and patterning during embryonic development at a finer resolution in vivo.

## Limitations

We have demonstrated the critical requirement of *olf413* for embryonic nervous system development and biosynthesis of neurotransmitter octopamine. But further qPCR and immunohistochemical quantification experiments are needed to be carried out to demonstrate if the *olf413* transcript and protein levels are affected in the mutants studied.

## Data Availability

Data available on request from the corresponding author.

## Abbreviations

SOG: Suboesophageal ganglion
CB: Central brain
VG: Ventral ganglion
BDGP: Berkeley Drosophila Genome Project
TβH: Tyramine β hydroxylase
GWA: Genome-wide association
VNC: Ventral nerve cord
BDSC: Bloomington Drosophila Stock Centre
BLAST: Basic Local Alignment Search Tool
VC: Ventral clusters
LC: Lateral clusters
DC: Dorsal clusters
PNS: Peripheral nervous system
KCs: Kenyon cells
CIMN: Centrointermedial neurons
DLN: Dorsolateral neurons
VS: Vertical system neurons
ORS: Optomotor responsive system
SM: Somatic muscle
MPC: Muscle precursor cells
FasII: Fasciclin II
ISN: Inter segmental neurons
SN: Segmental neurons
MH: Mouth hook
TR: Trachea
DIOPT: DRSC integrative ortholog prediction tool
DSHB: Developmental Studies Hybridoma Bank
